# Ultra-Short Cyclized β-Boomerang Peptides: Structures, Interactions with Lipopolysaccharide, Antibiotic Potentiator and Wound Healing

**DOI:** 10.3390/ijms24010263

**Published:** 2022-12-23

**Authors:** Sheetal Sinha, Vidhya Bharathi Dhanabal, Veronica Lavanya Manivannen, Floriana Cappiello, Suet-Mien Tan, Surajit Bhattacharjya

**Affiliations:** 1 School of Biological Sciences, Nanyang Technological University, 60 Nanyang Drive, Singapore 637551, Singapore; 2 Advanced Environmental Biotechnology Centre, Nanyang Environment and Water Research Institute, Nanyang Technological University, 1 Cleantech Loop, Singapore 637141, Singapore; 3 Interdisciplinary Graduate School, Nanyang Technological University, 50 Nanyang Avenue, Singapore 639798, Singapore; 4 Department of Biochemical Sciences, Laboratory Affiliated to Istituto Pasteur Italia-Fondazione Cenci Bolognetti, Sapienza University of Rome, 00185 Rome, Italy

**Keywords:** antibiotic potentiator, MDR Gram-negative bacteria, cyclic peptide, lipopolysaccharide (LPS)

## Abstract

Many antibiotics are ineffective in killing Gram-negative bacteria due to the permeability barrier of the outer-membrane LPS. Infections caused by multi-drug-resistant Gram-negative pathogens require new antibiotics, which are often difficult to develop. Antibiotic potentiators disrupt outer-membrane LPS and can assist the entry of large-scaffold antibiotics to the bacterial targets. In this work, we designed a backbone-cyclized ultra-short, six-amino-acid-long (WKRKRY) peptide, termed cWY6 from LPS binding motif of β-boomerang bactericidal peptides. The cWY6 peptide does not exhibit any antimicrobial activity; however, it is able to permeabilize the LPS outer membrane. Our results demonstrate the antibiotic potentiator activity in the designed cWY6 peptide for several conventional antibiotics (vancomycin, rifampicin, erythromycin, novobiocin and azithromycin). Remarkably, the short cWY6 peptide exhibits wound-healing activity in in vitro assays. NMR, computational docking and biophysical studies describe the atomic-resolution structure of the peptide in complex with LPS and mode of action in disrupting the outer membrane. The dual activities of cWY6 peptide hold high promise for further translation to therapeutics.

## 1. Introduction

Infections caused by antibiotic-resistant Gram-negative bacteria are significantly more challenging compared to those of Gram-positive infections [1,2,3,4,5]. Many frontline antibiotics are unable to mitigate Gram-negative bacterial infections, largely due to the permeability barrier of outer-membrane (OM) lipopolysaccharide (LPS). The outer leaflet of the outer membrane is rich in LPS, which forms an ordered structure establishing a permeability barrier for drug entry. The phosphate head groups in LPS bind to divalent cations (Ca^2+^ and Mg^2+^), whereas packing among the multi-acylated long lipid chains maintain the OM structure [6,7,8]. Discovery and development of new antimicrobials to combat infections of multi-drug-resistant (MDR) Gram-negative pathogens are highly needed [9,10,11]. A recent epidemiological study describing the global burden of bacterial antimicrobial resistance in 2019 observed that MDR Gram-negative bacteria, including *Escherichia coli*, *Klebsiella pneumoniae*, *Acinetobacter baumannii* and *Pseudomonas aeruginosa,* are a major cause of lethality [12]. This study pointed out an estimated 4.95 million deaths were associated with bacterial AMR in 2019. The emergence of carbapenem-resistant *K. pneumoniae*, *A. baumannii* and *P*. *aeruginosa* strains is causing hard-to-treat infections around the globe [13,14,15]. Despite certain toxicity, polymyxin B, a cyclic lipopeptide, and its derivatives are used to treat such infections [16,17].

Due to a lack of therapeutic modalities, antibiotic potentiators may provide an alternative solution for possible clinical use of large-scaffold inactive antibiotics against MDR Gram-negative pathogens [18,19,20,21,22]. In particular, antibiotic potentiators can disrupt the outer-membrane LPS permeability barrier, facilitating the entry of large-scaffold antibiotics to the intra-cellular bacterial targets [18,19]. Cationic peptides lacking antimicrobial activity or antimicrobial peptides (AMPs) are characterized with antibiotic potentiator activity. An archetypal example of an antibiotic potentiator is cyclic polymyxin B nonapeptide (PMN), a derivative of decapeptide PMB. PMN and its derivatives are devoid of any antimicrobial activity; however, these peptides act as antibiotic potentiators due to interactions with the negatively charged LPS outer membrane [23,24,25]. We designed, from the first principle, 12-amino-acid-long cationic/hydrophobic linear peptides for LPS interactions and antimicrobial and anti-endotoxic activity [26,27]. The designed peptides adopted a unique β-sheet structure in a complex with LPS micelle resembling a boomerang, hence, termed as β-boomerang peptides [26,27]. Structure–activity correlation studies of linear β-boomerang peptides delineated an eight-amino-acid (GWKRKRFG) -long peptide that can interact with LPS, assuming a well-defined folded structure. The eight-residue peptide is termed as the Structured LPS Binding Motif [27]. In this work, we prepare head–tail cyclized structured LPS binding motif peptide or cWY6 and extensively investigated this using NMR structural, biophysical, activity and toxicity assays. Our results demonstrate that the cWY6 peptide contains low hemolytic/cellular toxicity and can function as an antibiotic potentiator for several conventional antibiotics. In addition, cWY6 exhibits wound-healing activity in in vitro assays on human fibroblast and HaCaT cell lines. The atomic-resolution structure of cWY6 in LPS micelles delineates the mode of LPS outer-membrane recognition. Collectively, the ultrashort cWY6 peptide could be developed into antibiotic-based wound-healing therapeutics.

## 2. Results and Discussion

### 2.1. Design of cWY6 Peptide

The cWY6 peptide is designed based on antimicrobial and anti-endotoxic β-boomerang peptides. The design principle, structure and activity of 12-residue-long β-boomerang peptides are described in our previous works [26,27,28,29]. Briefly, the primary structure of a representative peptide, YVLWKRKRFIFI-amide, includes a stretch of four cationic residues (KRKR) at the centre of the 12-residue sequence. Residues at the N- and C-termini are either hydrophobic or aromatic with a propensity for β-sheet conformation. The central cationic residues of β-boomerang peptides interact with the negatively charged phosphate groups of LPS, whereas the hydrophobic/aromatic residues at the N- and C-termini are involved in packing or van der Waals interactions with the lipid A acyl chains of LPS [26,27]. The β-boomerang fold in complex with LPS micelle is stabilized by critical close π/π packings between residues W4 and F9 [27]. A shorter peptide, GWKRKRFG-amide, containing the central cationic motif KRKR and aromatic residues W and F assumed a compact folded structure in complex with LPS micelle [27]. The sequence WKRKRF is termed as Structured LPS Binding Motif. Here, we utilized the structured LPS binding motif peptide WKRKRY to design backbone cyclized cWY6 peptide. The Phe residue in the Structured LPS Binding Motif is replaced by Tyr due to superior antimicrobial activity of the 12-residue-long parent peptide [27]. The backbone cyclization is expected to stabilize the β-boomerang structure in free solution and also in complex with LPS outer membrane. Cyclization of bioactive peptides is an attractive strategy that would enhance activity and in vivo stability [30,31,32]. 

### 2.2. Atomic-Resolution Structures of cWY6 Peptide in Free Solution and in LPS Micelle

**Figure 1:**
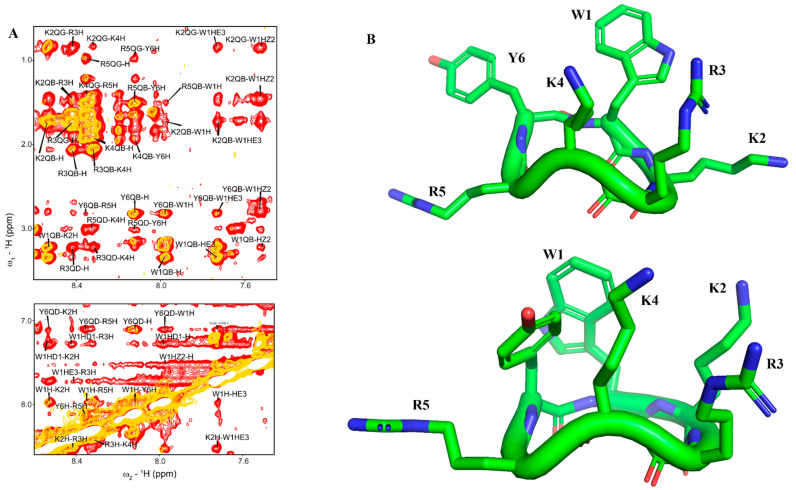
(**A**) Overlay of ^1^H−^1^H two-dimensional NOESY (yellow) and tr−NOESY (red) spectra of cWY6 in free solution (yellow) and in the presence of LPS micelle (red) showing NOEs among amide protons and aromatic resonances (bottom panel) and amide/aromatic resonances with aliphatic resonances (**upper** panel). (**B**) 3−D structures of cWY6 in free solution (**upper** panel) and in complex with LPS micelle (lower panel) represented by stick model. For cWY6, carbon atoms are in green, oxygen atoms are in red and nitrogen atoms are in blue.

We determine 3-D structures of cWY6 in free solution and in complex with LPS micelle. Sequence-specific resonance assignments of cWY6 were carried out by combined use of two-dimensional ^1^H-^1^H TOCSY and NOESY spectra. Figure 1A shows the overlay of selected regions of NOESY spectra of cYW6 in free solution (yellow contour) and in the presence of LPS micelle (red contour). There are more NOEs observed in complex with LPS, indicating compact conformations in the cWY6 peptide. Free and LPS-bound structures of the peptide are obtained from CYANA using NOE-driven distance constrains and dihedral-angle constrains (see methods). Figure 1B shows NMR-derived structures of cYW6 in free solution (upper panel) and in complex with LPS (lower panel).

Table 1 summarizes structural statistics and constraints used in structure calculations. The LPS-bound structure of cWY6 is better defined compared to that in free solution, as indicated by the lower RMSD value of 0.52 Å vs. 1.14 Å distance constrains. 

**Figure 2:**
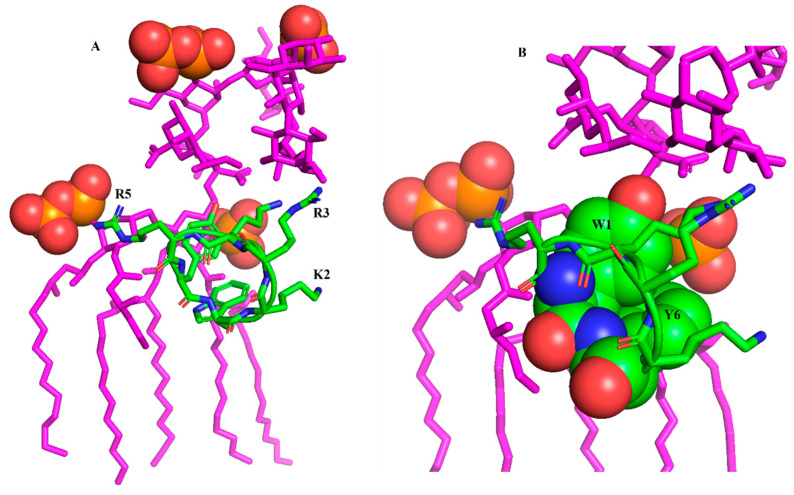
(**A**) Docked complex of cWY6 and LPS (magenta) showing potential molecular interactions. The sidechains of cationic residues R5, R3 and K2 of cWY6 are in proximity to negatively charged phosphate groups (red, space filled) of lipid A of LPS. (**B**) An expanded view of the docked complex of cWY6/LPS highlighting packing of the aromatic sidechains of residues Trp1 and Tyr6 with the acyl chains of LPS. For cWY6, carbon atoms are in green, oxygen atoms are in red and nitrogen atoms are in blue.

Further, the LPS-bound structure of the cWY6 peptide is docked with LPS, realizing potential interactions between the peptide and LPS outer membrane (Figure 2).

As seen, the central cationic residues and aromatic residues of the cWY6 are engaged in interactions with outer-membrane LPS. In particular, the bis-phosphate groups of lipid A in LPS may form ionic interactions with the guanidinium sidechains of residues R3 and R5 (Figure 2A). Further, the sidechain of residue K2 can be involved in ionic interactions with the phosphate of lipid A (Figure 2A). The aromatic sidechains of residues W1 and Y6 demonstrate close packing with acyl chains of LPS (Figure 2B). The mode of binding of cYW6 peptide with LPS could be pivotal in disrupting the permeabilization barrier of the outer membrane of Gram-negative bacteria. 

### 2.3. Outer-Membrane Permeabilization and LPS Interactions of cWY6 Peptide 

**Figure 3:**
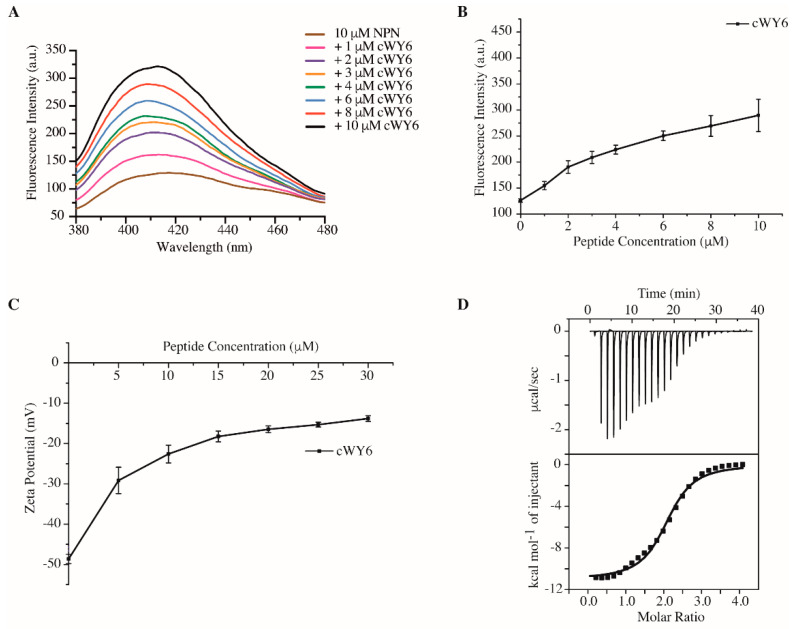
(**A**) Fluorescence emission spectra of NPN in solutions containing *E. coli* cells at increasing concentrations of cWY6 peptide. (**B**) Plot showing increase in fluorescence intensity at the emission maxima of NPN as a function of concentration of cWY6 peptide. (**C**) Plot showing zeta potential values of *E. coli* cell solutions containing increasing concentrations of cWY6 peptide. (**D**) ITC thermogram of interactions between cWY6 peptide and LPS. The top panel of ITC plot shows titrated peaks as function of time, whereas the lower panel delineates data fitting of integrated heat values at each point.

We carried out 1-N-phenylnaphthylamine (NPN) uptake assays, determining the outer-membrane permeabilization activity of the cYW6 peptide. NPN is a fluorogenic probe, which shows high-intensity emission only in non-polar membrane environments. NPN showed an increased fluorescence emission intensity upon additions of cWY6 in solutions of *E. coli* cells in a dose-dependent manner (Figure 3A). As seen, cWY6 caused a continuous increase in fluorescence intensity of the NPN probe (Figure 3B).

Figure 3C delineates zeta-potential measurements of *E. coli* cells at various concentrations, 5 to 30 μM, of the cWY6 peptide. Peptide additions caused a significant decrease in the zeta-potential values of *E. coli* cell solutions. The data suggest that the cWY6 peptide is able to neutralize the surface charge of Gram-negative bacteria. Further, interactions and thermodynamics of cWY6 peptide binding to LPS are quantified by isothermal titration calorimetry (ITC) experiments (Figure 3D). LPS–peptide interaction is exothermic in nature, as indicated by negative heat change or downward ITC peaks. A dissociation constant (K_d_) value of 3.03 μM was estimated from the ITC data for the LPS/cWY6 complex. Therefore, the aforementioned data demonstrate that the designed cWY6 peptide is able to disrupt integrity of the outer membrane and can neutralize the surface charge of Gram-negative bacteria. Further, cWY6 confers binding to LSP micelle, as demonstrated by ITC. 

### 2.4. Interactions of cWY6 Peptide with LPS and Liposomes

**Figure 4:**
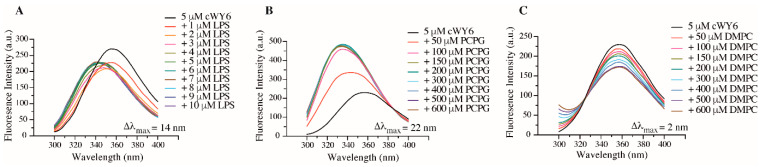
Fluorescence emission spectra of Trp residue of cWY6 peptide at increasing concentrations of (**A**) LPS micelle, (**B**) POPC/POPG 3:1 liposomes and (**C**) DMPC liposomes.

Intrinsic Trp fluorescence is utilized to determine the binding specificity of cWY6 with negatively charged and zwitterionic lipids (Figure 4).

In these separate experiments, cWY6 peptide samples were titrated with LPS micelles (Figure 4A), negatively charged liposome 1-palmitoyl-2-oleoyl-sn-glycero-3-phosphocholine (POPC):1-Palmitoyl-2-Oleoyl-sn-Glycero-3-Phosphoglycerol (POPG) (3:1) (Figure 4B) and liposomes consisted of zwitterionic lipids 1,2-dimyristoyl-*sn*-glycero-3-phosphocholine (DMPC) (Figure 4C). As expected, the cWY6 peptide selectively interacted only with negatively charged lipids and liposomes. Emission maxima of Trp residue of the peptide delineated a dramatic blue shift, either in the presence of LPS micelle (Figure 4A) or in POPC:POPG liposome (Figure 4B). Such spectral changes are diagnostic of insertion of the Trp residue from the cWY6 peptide into the non-polar milieu of the lipids. By contrast, limited spectral changes of Trp can be observed in titrations with DMPC liposomes (Figure 4C), suggesting probable surface binding of the peptide. 

### 2.5. Perturbation of Higher-Order Aggregates of LPS by cWY6 Peptide

**Figure 5:**
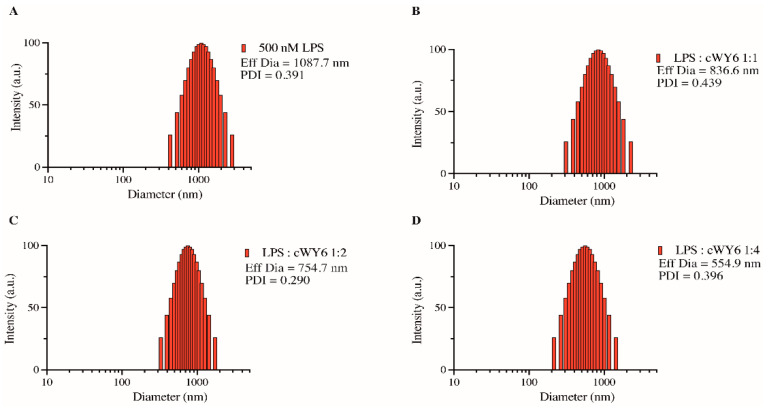
Bar diagram, obtained from dynamic light scattering experiments, showing effective diameter of LPS micelle alone (**A**) and at various ratios of LPS/cWY6 peptide (**B**–**D**).

We next examined the ability of the designed cyclic peptide, cWY6, to cause any detectable changes in LPS-aggregated structures. It may be noted that LPS forms high-molecular-weight aggregated micelles of heterogeneous size distribution in solution [26,27]. AMPs and LPS binding peptides are known to dissociate aggregated structures of LPS into smaller sizes, which are often correlated with antimicrobial and antiendotoxic activity [26,27]. Size distribution and effective diameter of LPS in free solution and in the presence of the cWY6 peptide at different molar ratios are obtained from dynamic light scattering (DLS) experiments (Figure 5). As is evident, LPS in free solution forms large aggregates with an effective diameter of 1087.7 nm (Figure 5A). Inclusions of the cWY6 peptide in solutions of LPS at 1:1 (Figure 5B), 1:2 (Figure 5C) and 1:4 (Figure 5D) ratios demonstrate a significant decrease in the effective diameter of LPS micelle. These data suggest that binding of the cWY6 peptide to LPS micelle imparts substantial destabilization of the aggregated structures of LPS, rendering dissociation into smaller-size particles. 

### 2.6. Red Blood Cell (RBC) Lysis and Mammalian Cell Toxicity of cWY6 Peptide

**Figure 6:**
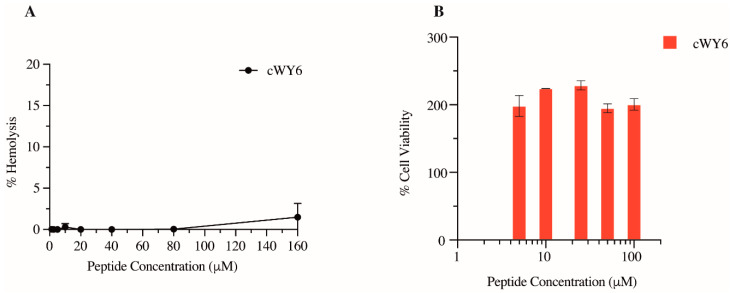
(**A**) Plot showing % hemolysis of RBC as a function of concentrations of cWY6 peptide. (**B**) Bar diagram showing % cell viability of mouse fibroblast cells determined from MTT assays as a function of concentrations of cWY6 peptide.

The potential cytotoxicity of the designed cyclic cWY6 peptide was evaluated by hemolysis and MTT assays. Figure 6A shows % hemolysis of human RBC at different peptide concentrations. Clearly, the hemolytic activity of the cWY6 peptide is highly limited since only <5% hemolysis was found at the highest peptide concentration of 160 μM. Subsequently, via MTT assays, we assessed cWY6 peptide toxicity on mouse fibroblast NIH3T3 cells (Figure 6B). 

As shown, cWY6 the peptide did not cause cell death at any tested concentration, ranging from 5 to 100 μM. Interestingly, we observed that cell viability increased in the presence of the cWY6 peptide (Figure 6B). This result could indicate the peptide’s ability to induce cell proliferation. Such activity can give rise to a potential wound-healing function of the peptide.

### 2.7. In Vitro Wound-Healing Activity of cWY6 Peptide

**Figure 7:**
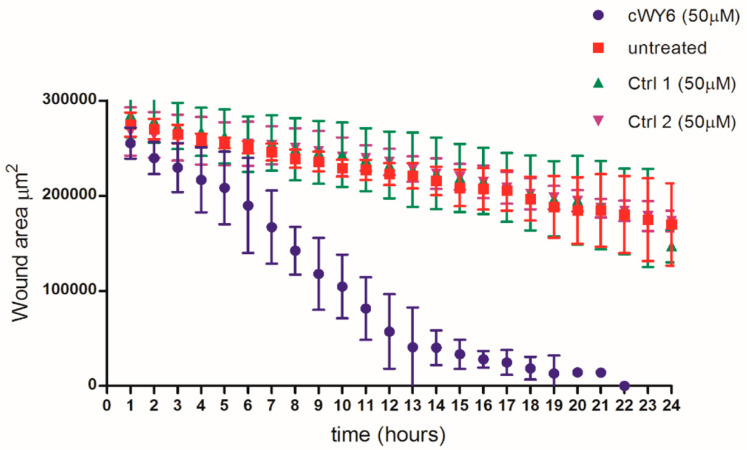
In vitro pseudo-wound-healing effect of cWY6 peptide (blue line) on BJ fibroblast cells. For comparison, wound-healing assays were also carried out with two analogs of cWY6 (green and purple lines) and untreated control (red line). Ctrl 1 (AKRKRA) and Ctrl 2 (WDDDY) are cyclized analogs of cWY6.

Observation of cell proliferation of mouse fibroblast cells prompted us to further assess any potential wound-healing property in the peptide. Wound-healing assays were performed using BJ human fibroblast and HaCaT keratinocyte cell lines (see Methods). Figure 7 compares the time-course analysis of pseudo-wound closure of human BJ fibroblast cells using 50 μM cWY6 peptide or two peptide analogs of cWY6.

**Figure 8:**
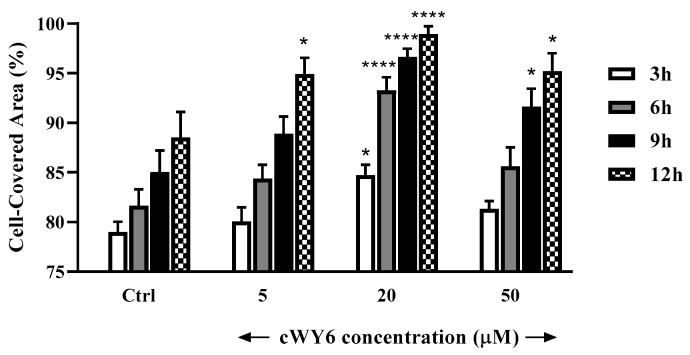
In vitro pseudo-wound-healing effect of cWY6 peptide at different concentrations on HaCaT cells. The percentage of cell-covered area at each time point is reported on the *y*-axis. Control (Ctrl) is given by vehicle-treated cells. All data are the mean of three independent experiments ± standard error of the mean (SEM). The levels of statistical significance between Ctrl and peptide-treated samples are indicated as follows: * *p* < 0.05 and **** *p* < 0.0001.

The designed peptide cWY6 was able to induce cell migration, causing a significantly faster closure of the gap in the pseudo-wound-healing assay, while the two cyclic peptide analogs, AKRKRA (Ctrl 1) and WDDDY (Ctrl 2), did not show any significant difference compared to the untreated control. This result suggests the involvement of aromatic (Trp and Tyr) and central cationic residues in cell-migration activity (Figure 7). Moreover, the cWY6 peptide also promoted HaCaT cell migration, as indicated by its ability to induce about 100% coverage of the pseudo-wound within 12 h at an optimal concentration of 20 μM (Figure 8).

### 2.8. Antibiotic Potentiator Activity of cWY6 Peptide

The designed cWY6 peptide displayed weak antibacterial activity against Gram-negative strains of *Escherichia coli* (Migula) ATCC 25922, drug-resistant *Acinetobacter baumannii* ATCC BAA-1798, *Pseudomonas aeruginosa* ATCC 27853, *Klebsiella pneumoniae* ATCC 13883 and *Salmonella enterica* ATCC 14028. We further investigated the antibiotic potentiator activity of cWY6 for vancomycin, rifampicin, novobiocin and erythromycin against Gram-negative bacteria. Minimal Inhibitory Concentration (MIC) values of antibiotic and peptide combinations were estimated via checkerboard analysis (see Materials and Methods). 

**Figure 9:**
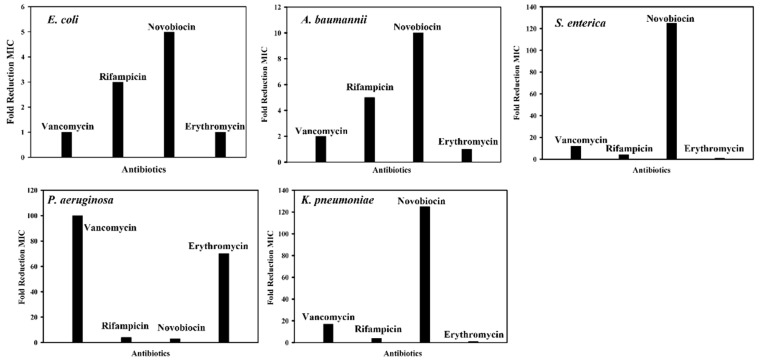
Bar diagrams showing fold reduction in MICs of antibiotics of grown inhibition of Gram-negative bacteria in the presence of 16 μM cWY6 peptide.

Figure 9 shows the fold reduction in MIC of antibiotics in the presence of 16 μM cWY6 peptide against *E. coli* (Migula) ATCC 25922, drug-resistant *A. baumannii* ATCC BAA-1798, *S. enterica* ATCC 14028, *P. aeruginosa* ATCC 27853 and *K. pneumonia* ATCC 13883. Interestingly, the cWY6 peptide demonstrated antibiotic potentiator activity that is specific to bacterial strains and antibiotics. The fold reduction in MIC values of antibiotics against *the E. coli* strain appears to be limited, 3- to 5-fold. Only a moderate reduction in MIC can be observed for rifampicin and novobiocin. A similar trend is observed for vancomycin and rifampicin against drug-resistant *A. baumannii*. However, the MIC of novobiocin is estimated to be reduced by 10-fold in the presence of the cWY6 peptide. A strong synergy between cWY6 and novobiocin is detected for *S. enterica and K. pneumonia*, whereby MIC values of novobiocin are reduced more than 120-fold. cWY6 demonstrates higher potentiation of antibiotics vancomycin and erythromycin against *P. aeruginosa*. 

## 3. Materials and Methods

### 3.1. Peptide Synthesis and Chemicals

cWY6 and analog peptides were purchased from GL Biochem (Shanghai, China), with >95% purity. Molecular weight of the peptides was confirmed by mass spectrum analysis. All chemicals used in the work were of analytical grade. 

### 3.2. NMR Studies, Structure Calculation and LPS Docking of cWY6 Peptide

Two-dimensional ^1^H-^1^H TOCSY (mixing time 70 ms) and ^1^H-^1^H NOESY spectra (mixing time 200 ms) of cWY6 peptide, 300 μM concentration, were acquired in 10 mM sodium phosphate buffer, pH 5.0, concentration, at 298 K using a Bruker 600 MHz spectrometer (Billerica, MA, USA) equipped with cryoprobe. Two-dimensional ^1^H-^1^H tr-NOESY spectra (mixing time 100 ms) of cWY6 peptide were collected in 10 mM sodium phosphate buffer, pH 5.0, 298 K, containing 20 μM LPS (*E. coli* O111:B4, Sigma Aldrich, St. Louis, MI, USA). NMR spectra were analyzed using SPARKY (T. D. Goddard and D.G. Kneller, University of California, San Francisco, CA, USA). Atomic-resolution structures of cYW6 peptide in free solution and in LPS micelle were determined by CYANA [33] using NOE-driven distance and backbone dihedral-angle constrains. Typically, based on intensity (strong, medium and weak), NOE cross-peaks were translated into upper-bound distances of 2.5, 3.5 and 5.0 Å, respectively. Dihedral-angle constrains of amino acid residues were obtained from PREDITOR web server using C^a^H secondary chemical shift [34]. LPS-bound structure of cWY6 peptide was docked onto LPS (pdb 1QFG) by PatchDock program, 1.3 version [35]. The lowest-energy structure of LPS/peptide complex is used for analysis.

### 3.3. NPN, z Potential and ITC Experiments 

Fluorescence emission spectra of NPN (10 μM) were obtained using a Cary Eclipse spectrophotometer (Hitachi, Tokyo, Japan), either in absence or in the presence of *E. coli* of cell density OD_600_ 0.5 from a mid-log phase culture in 10 mM sodium phosphate buffer. Aliquots of cWY6 peptide were added to *E. coli* cell suspension to detect effects on NPN fluorescence. The probe was excited at a wavelength of 350 nm and emission was recorded from 390 to 450 nm wavelength. For ζ-potential experiments, *E. coli* cell density was adjusted to an OD_600_ of 0.2 in LB media. ζ-potential of *E. coli* cell suspension was determined alone followed by additions of cWY6 peptide at increasing concentrations. For each data point, 3 measurements of 100 runs each were performed. The experiments were carried out on a zeta sizer Nano ZS (Malvern Instruments, Worcestershire, UK) equipped with a 633 nm He laser. Binding interactions of cWY6 peptide with LPS were determined by use of ITC (Microcal, ITC 200, Northampton, MA, USA) experiments in 10 mM sodium phosphate buffer, pH 7.0 at 298 K. Typically, aliquots of LPS (50 μM) were injected from syringe into sample cell containing 1 μM cWY6 peptide. Samples were stirred at a speed of 900 rpm. Interaction parameters were obtained from a single-site binding model. 

### 3.4. Intrinsic Trp Fluorescence of cWY6 Peptide in LPS and Liposomes 

Intrinsic Trp fluorescence experiments of cWY6 were conducted for cWY6 peptide in a Cary Eclipse fluorescence spectrophotometer using a 0.1 cm-path-length quartz cuvette. Samples were citated at a wavelength of 280 nm and emission spectra were recorded from 300 to 400 nm. In these experiments, 5 μM of cWY6 peptide in 10 mM sodium phosphate buffer, pH 7.0, was titrated at increased concentrations of LPS, POPC:POPG (3:1) and DMPC liposomes. Liposomes were prepared based on the method published elsewhere [36].

### 3.5. Dynamic Light Scattering (DSL) Studies of LPS Micelles and cWY6 

Ability of the cWY6 peptide to perturb LPS micelles was examined by analyzing the particle size of 500 nM LPS in the presence of increasing peptide concentrations. The mean diameter of free LPS was first determined in 10 mM phosphate buffer, pH 7, using a dynamic light scattering instrument (Brookhaven Instruments Corp., Holtsville, NY, USA). DLS measurements were then made for LPS:peptide ratios of 1:1, 1:2 and 1:4. The scattering data were analyzed using the particle-sizing software provided with the instrument.

### 3.6. Cell Lines and In Vitro Wound-Healing Assay

The human BJ skin fibroblast cell line was obtained from the American Type Culture Collection and maintained in Minimum Essential Medium (MEM) containing 10% heat-inactivated fetal bovine serum (FBS) and supplemented with 100 U/mL of penicillin and 100 μg/mL streptomycin. 

The human immortalized keratinocyte HaCaT cells were purchased from AddexBio (San Diego, CA, USA). HaCaT cells were cultured in Dulbecco’s modified Eagle’s medium containing 4 mM glutamine, 10% FBS and 0.1 mg/mL of penicillin and streptomycin, at 37 °C and 5% CO_2_. For fibroblast cell line, 2D wound-healing experiments were performed as described previously [37] with modifications. Then, 1.5 × 10^4^ cells were seeded into each chamber of a two-well culture insert (Ibidi GmbH, Martinsried, Germany) that was placed on a non-coated well of a 12-well culture plate followed by overnight culture in a humidified CO_2_ incubator. The culture insert was then removed. Fresh medium (with or without peptides) was added to well, and time-lapse images of gap closure were recorded on a live-cell imaging system (Olympus IX83, UPLFLN-PH, Olympus) followed by analyses using ImageJ software Advanced Imaging Facilities, University of Leicester, Leicester, UK). 

For wound-healing assay on keratinocytes, about 4 × 10^4^ HaCaT cells were seeded in each side of an ibidi culture insert and grown to confluence at 37 °C and 5% CO_2_, as previously described [38,39]. Afterwards, cells were treated with cWY6 peptide at different concentrations, as indicated. At the time of insert removal (0 h) and after 3, 6, 9 and 12 h from peptide addition, cells were checked for migration under an inverted microscope (Olympus CKX41) at × 4 magnification and photographed with a Color View II digital camera. The percentage of cell-covered area was calculated by WIMASIS Image Analysis program. https://www.wimasis.com/en/).

### 3.7. Antibiotic Potentiator Activity of cWY6 Peptide

The MICs of antibiotics and cWY6 were measured using the broth-dilution method. Antibiotic potentiator activity of the peptide was determined from checkerboard assays [40]. Single colony of five Gram-negative bacteria strains (*Escherichia coli(Migula)* ATCC 25922, *Pseudomonas aeruginosa* ATCC 27853, *Salmonella enterica* ATCC 14028, *Klebsiella Pneumoniae* ATCC 13883 and *Acinetobacter baumannii* ATCC BAA-1798) obtained from LB plate were inoculated in 5 mL of LB broth to prepare overnight starter cultures. The mid-log cultures were then prepared by diluting the overnight starter cultures by 1:100 in MH broth, followed by incubation at 37 °C, 183 RPM for 3 h. The mid-log cultures were further diluted with MH broth to an OD_600_ value of 0.002. Final OD of mid-log culture would be 0.001 when 50 μL of culture is added to 50 μL of peptide–antibiotic mixture. Stocks of cWY6 and different antibiotics were prepared with MilliQ water. In a 96-well microtiter plate, 50 μL of peptide–antibiotic mixture with peptide concentration ranging from 0–64 μM and antibiotic concentration ranging from 0–128 μM was added. Then, 50 μL of diluted bacteria culture was included to each well to obtain a final volume of 100 μL. In separate wells, positive control consisting of 50 μL Milli-Q water and 50 μL diluted bacteria culture and negative control consisting of 100 μL MH broth was prepared. The microtiter plates were incubated at 37 °C for 18 h and OD_600_ value was measured using a BioTek Cytation 5 Imaging Reader (Agilent, Santa Clara, CA, USA).

### 3.8. RBC Lysis and Cell Viability Assays

RBC lysis and MTT assays were conducted as described in our previous work [36]. Fresh human blood samples, obtained from a healthy donor, were washed with PBS (10 mM sodium phosphate buffer, 150 mM NaCl, pH 7) and diluted in PBS to 5%. Lysis assays were carried out in 96-well plates, whereby 50 μL of varying concentrations of cWY6 was prepared followed by additions of 50 μL of 5% blood sample to each well. The assay plates were incubated for 1 h at 37 °C. Blood samples were centrifuged and the OD_540_ of the supernatant was measured. Blood samples were also treated with 1% triton-X serving as positive control and PBS was taken as negative control. Standard MTT assays of cell viability was used to estimate effect of cWY6 peptide on NIH3T3 mouse cell line. Typically, in a 96-well plate, 10,000 cells were added in each well and 100 μL of cWY6 peptide of varying concentrations was added with fresh media. The plates were incubated for 4 h at 37 °C and 5% CO_2_. Further, 10 μL of MTT reagent was added in each well followed by incubation for 4 h at 37 °C and 5% CO_2_. Finally, media were removed from every well and 100 μL of DMSO was added to solubilize the dye crystals followed by measuring OD at 540 nm. For negative control, no peptide was added and for positive control, no cells were added. The % cell viability was estimated by: % Cell Viability = $\frac{\mathrm{ODnegative}-\mathrm{OD}}{\mathrm{ODnegative}-\mathrm{ODpositive}}$ * 100, where OD: OD_540_ for sample well, OD_negative_: OD_540_ for negative control, OD_positive_: OD_540_ for positive control.

### 3.9. Statistical Analysis

Quantitative data derived from independent experiments were expressed as the mean ± standard error of the mean (SEM). Statistical significance was determined using two-way analysis of variance (ANOVA) with PRISM software 7.0 (GraphPad, San Diego, CA, USA). Differences were considered statistically significant for a *p* value of <0.05. 

## 4. Conclusions

Cyclic peptides or peptide macrocycles offer an exciting avenue for therapeutic developments due to their high stability and improved biological activity. In this work, we design an ultrashort cyclic peptide, cWY6, based on the “structured LPS binding motif” of β-boomerang peptides. The cycle peptide interacted with LPS and permeabilizes the outer membrane, causing the potentiation of several conventional antibiotics, killing Gram-negative bacteria, including drug-resistant strains. LPS, lipid interactions and atomic-resolution structural data in the LPS outer-membrane of the cWY6 peptide provided mechanistic insights into membrane permeabilization. In addition, the cWY6 peptide is also endowed with wound-healing activity in in vitro assays and is poorly hemolytic and cytotoxic. Therefore, the functional attributes and structural details of the designed cWY6 peptide described here could be useful for developing novel antibiotics and wound-healing therapeutics. 

## Figures and Tables

**Table 1:** Summary of structural statistics: 1, cWY6 in free solution; 2, cWY6 in presence of LPS.

	1	2
**Distance constraints**		
Sequential [|i − j| = 1]	19	36
Medium range [1 < |i − j| < 4]	2	7
Long range [|i − j| ≥ 4]	4	16
Total	53	96
**Dihedral-angle constraints**	8	8
**Deviation from mean structure (Å)**		
All heavy atoms	1.14	0.52
**Ramachandran plot for the mean structure (%residues)**		
Most favoured region	75	50
Additionally allowed region	25	50
Generously allowed region	0	0
Disallowed region	0	0

## Data Availability

The original contributions presented in the research are included in the article; further inquiries can be directed to the corresponding author.
